# A Conservative Assessment of the Major Genetic Causes of Idiopathic Chronic Pancreatitis: Data from a Comprehensive Analysis of *PRSS1*, *SPINK1*, *CTRC* and *CFTR* Genes in 253 Young French Patients

**DOI:** 10.1371/journal.pone.0073522

**Published:** 2013-08-08

**Authors:** Emmanuelle Masson, Jian-Min Chen, Marie-Pierre Audrézet, David N. Cooper, Claude Férec

**Affiliations:** 1 Institut National de la Santé et de la Recherche Médicale, U1078, Brest, France; 2 Laboratoire de Génétique Moléculaire et d’Histocompatibilité, Centre Hospitalier Régional Universitaire Brest, Hôpital Morvan, Brest, France; 3 Etablissement Français du sang – Bretagne, Brest, France; 4 Institute of Medical Genetics, School of Medicine, Cardiff University, Cardiff, United Kingdom; 5 Faculté de Médecine et des Sciences de la Santé, Université de Bretagne Occidentale, Brest, France; Centro Nacional de Investigaciones Oncológicas (CNIO), Spain

## Abstract

Idiopathic chronic pancreatitis (ICP) has traditionally been defined as chronic pancreatitis in the absence of any obvious precipitating factors (e.g. alcohol abuse) and family history of the disease. Studies over the past 15 years have revealed that ICP has a highly complex genetic architecture involving multiple gene loci. Here, we have attempted to provide a conservative assessment of the major genetic causes of ICP in a sample of 253 young French ICP patients. For the first time, conventional types of mutation (comprising coding sequence variants and variants at intron/exon boundaries) and gross genomic rearrangements were screened for in all four major pancreatitis genes, *PRSS1*, *SPINK1*, *CTRC* and *CFTR*. For the purposes of the study, synonymous, intronic and 5'- or 3'-untranslated region variants were excluded from the analysis except where there was persuasive evidence of functional consequences. The remaining sequence variants/genotypes were classified into causative, contributory or neutral categories by consideration of (i) their allele frequencies in patient and normal control populations, (ii) their presumed or experimentally confirmed functional effects, (iii) the relative importance of their associated genes in the pathogenesis of chronic pancreatitis and (iv) gene-gene interactions wherever applicable. Adoption of this strategy allowed us to assess the pathogenic relevance of specific variants/genotypes to their respective carriers to an unprecedented degree. The genetic cause of ICP could be assigned in 23.7% of individuals in the study group. A strong genetic susceptibility factor was also present in an additional 24.5% of cases. Taken together, up to 48.2% of the studied ICP patients were found to display evidence of a genetic basis for their pancreatitis. Whereas these particular proportions may not be extrapolable to all ICP patients, the approach employed should serve as a useful framework for acquiring a better understanding of the role of genetic factors in causing this oligogenic disease.

## Introduction

Chronic pancreatitis, a persistent inflammation of the pancreas that results in irreversible morphological changes and impairment of both exocrine and endocrine functions, is a potentially life-threatening disease. In Western countries, alcohol abuse is the leading cause of chronic pancreatitis, accounting for approximately 70% of all cases. Other aetiological factors, including hyperparathyroidism, hypertriglyceridemia, duct obstruction, trauma, pancreas divisum, autoimmune pancreatitis and hereditary pancreatitis, are responsible for causing the disease in approximately 10% of patients. The remaining ~20% of cases, in which none of the abovementioned aetiological factors can be identified, have been collectively termed idiopathic chronic pancreatitis (ICP) [1].

Over the past 15 years, the importance of genetic factors in the aetiology of ICP has been increasingly recognized. This began with the report of an association between *CFTR* (cystic fibrosis transmembrane conductance regulator; MIM #602421) mutations and ICP in 1998 [2,3]. In the human exocrine pancreas, CFTR is predominantly expressed at the apical plasma membrane of the ductal and centroacinar cells that line the small pancreatic ducts, and controls cAMP-stimulated HCO_3_^−^ secretion into the duct lumen [4–6]. The major function of CFTR in the pancreas is to dilute and alkalinize the protein-rich acinar secretions, thereby preventing the formation of protein plugs that predispose to pancreatic injury [7,8]. Whereas the presence of two highly deleterious (‘severe’) *CFTR* alleles (as in p.F508del homozygotes) is necessary to give rise to classic cystic fibrosis, heterozygosity for such an allele is sufficient to confer an increased risk of ICP, whilst compound heterozygosity, involving a severe *CFTR* allele plus a less deleterious (‘mild’) allele (e.g. p. F508del/p.R117H), confers a further increase in risk [7,8]. Since cystic fibrosis carriers (i.e. individuals harbouring a heterozygous severe *CFTR* allele) correspond to 3% of the general population in many European countries, *CFTR* mutations represent a major risk factor for ICP [7,8].

Gain-of-function *PRSS1* (encoding cationic trypsinogen; MIM #276000) missense mutations were first reported in individuals with ICP in 1999 [9,10], three years after the identification of *PRSS1* as a causative gene for hereditary pancreatitis [11]. In addition to gain-of-function missense mutations, gain-of-function *PRSS1* gene duplication and triplication copy number mutations have also been found in ICP patients [12]. Finally, and most importantly, the involvement of *PRSS1* contributed directly to the identification of two further chronic pancreatitis susceptibility genes, *SPINK1* (encoding pancreatic secretory trypsin inhibitor; MIM #167790) [13] and *CTRC* (encoding chymotrypsin C, a trypsin-degrading enzyme [14]; MIM #601405) [15,16]. Given that in the pancreas, *PRSS1*, *SPINK1* and *CTRC* are expressed exclusively in the acinar cells, these genetic findings suggested an important role for prematurely activated trypsinogen within the pancreatic acini in initiating chronic pancreatitis [8,17,18].

The abovementioned four genes represent the most extensively studied chronic pancreatitis-causing (or predisposing) genes identified to date. However, only very recently were they all analysed together in a single study of ICP [19]. Such an analysis is essential for an assessment of each gene’s relative contribution to the aetiology of the disease and to reveal the potential interactions between the different genes/gene products. However, Rosendahl et al. [19] included both ICP and hereditary pancreatitis patients in their study and only screened for micro-lesions (not gross rearrangements) in the four genes. In addition, they focused on the overall contribution of *CFTR* variants to the aetiology of chronic pancreatitis at the population level. In the current study, we have performed a comprehensive mutation screening analysis of all four known pancreatitis genes in a relatively large and clinically homogenous sample of French ICP patients. In contrast to the Rosendahl study [19], we opted to assess the pathogenic relevance (i.e. causative or contributory) of the detected variants/genotypes to their respective carriers. This strategy allowed us to assign genetic causality in a significant fraction of our ICP patients.

## Materials and Methods

### Ethics statement

This study was approved by the Ethical Review Committee of the Université de Bretagne Occidentale. All participating patients provided informed consent for genetic analysis.

### Patients

In accordance with our previous studies [12,16], ICP was defined as chronic pancreatitis in the absence of any obvious precipitating factors (e.g. alcohol abuse, infection or trauma) and in the absence of a positive family history; whilst “young” ICP patients were defined as those patients in whom the age of disease onset was known to be ≤20 years or in whom the diagnosis of chronic pancreatitis was made before the age of 20. A total of 253 young French ICP patients were included in this study; they represent those young ICP patients who were recruited from both public and private clinics/hospitals in mainland France between February 1998 and July 2007 and whose DNA samples were available for the mutational analysis of the *CFTR*, *PRSS1*, *SPINK1* and *CTRC* genes. Most of the patients have been previously reported [12,16].

### Screening for point mutations and micro-insertions/micro-deletions in the four major pancreatitis genes

All exons and exon/intron junctions of the *PRSS1* and *SPINK1* genes were analysed using previously described denaturing high-performance liquid chromatography methods [20,21].

Exons and exon/intron junctions of the *CFTR* gene were screened by either denaturing high-performance liquid chromatography [22] and/or high-resolution DNA melting analysis [23]. Additionally, the *CFTR* intron 8 poly(T) variants were analysed according to the method of Friedman and colleagues [24] whilst the poly(TG) repeat number in individuals carrying the *CFTR* intron 8 5T allele was assessed using the fluorescent multiplex PCR method of Mantovani et al. [25].

All exons of the *CTRC* gene and their immediate flanking sequences were analysed by direct sequencing as previously described [16].

### Screening for gross genomic rearrangements or copy number variation in the four major pancreatitis genes

Gross genomic rearrangements or copy number mutations in the four major pancreatitis genes were sought by means of quantitative fluorescent multiplex-PCR as described elsewhere [16,26–28].

### Principles for variant/genotype classification

**Figure 1:**
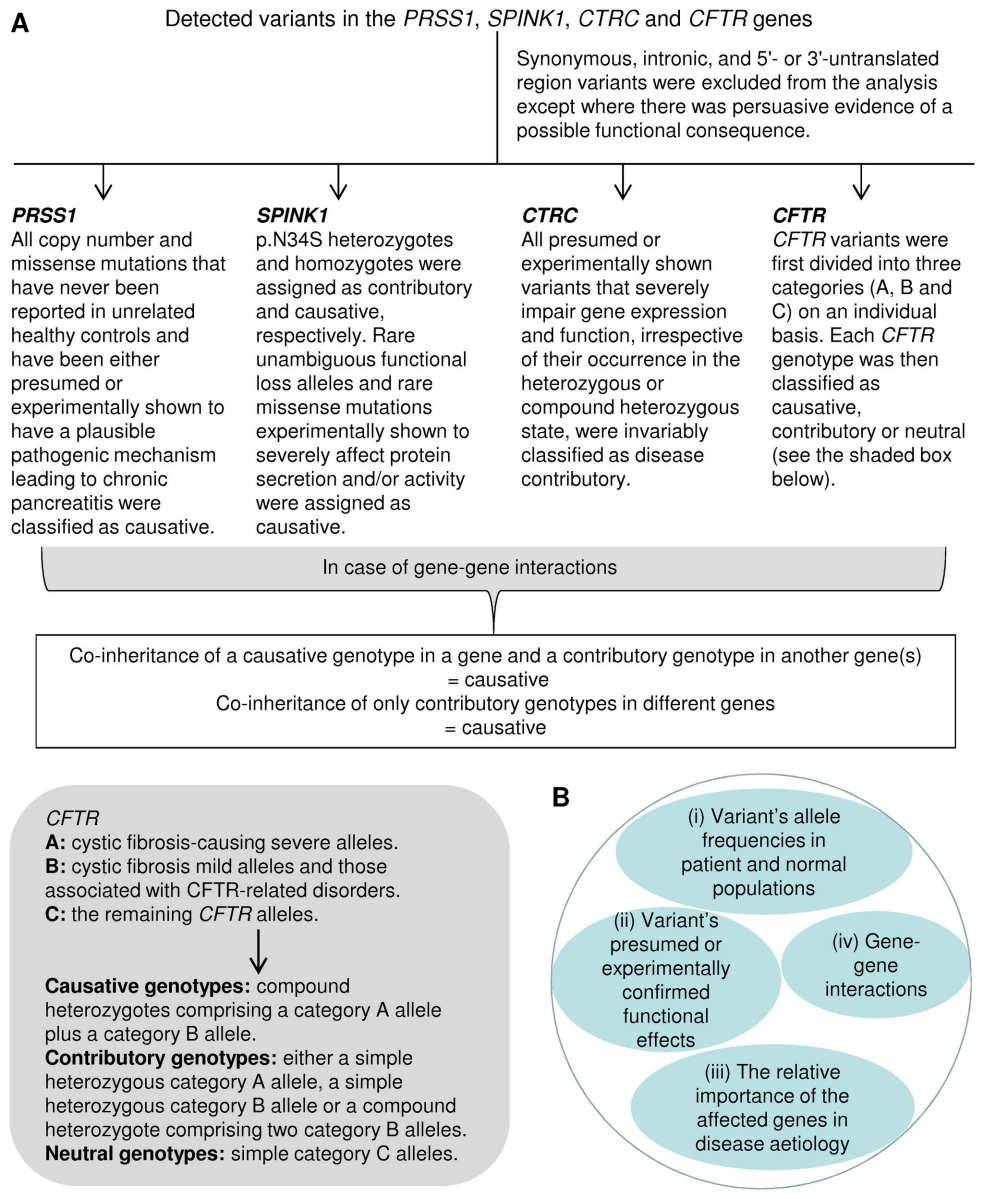
Principles of variant classification in terms of their pathogenic relevance. See Results and Discussion for details.

To provide a conservative assessment of the extent of the genetic basis of ICP in the studied subjects and for the sake of simplicity, certain variants of unknown functional significance were first excluded from the analysis (Figure **1A**). The remaining sequence variants/genotypes were then sub-classified into causative, contributory or neutral categories (Figure **1A**) through consideration of four specific factors (Figure **1B**).

## Results and Discussion

### Characteristics of the patient cohort

The patient cohort comprised 253 subjects and was characterized by two features. First, it included only subjects with clinically well-defined ICP as ascertained by experienced clinicians. Second, it included only young patients (age at diagnosis or age of onset ≤20 years). A major advantage of employing this homogeneous patient sample for genetic analysis is that interference from potential confounding factors should be significantly reduced.

### Mutation data analysis and classification

This study is the first to have analysed both intragenic mutations and gross genomic rearrangements in all four genes in a group of ICP patients. Apart from the *PRSS1* gene duplication and triplication copy number mutations, we did not identify any further gross rearrangements in any of the four genes in the studied cohort (see below).

**Figure 2:**
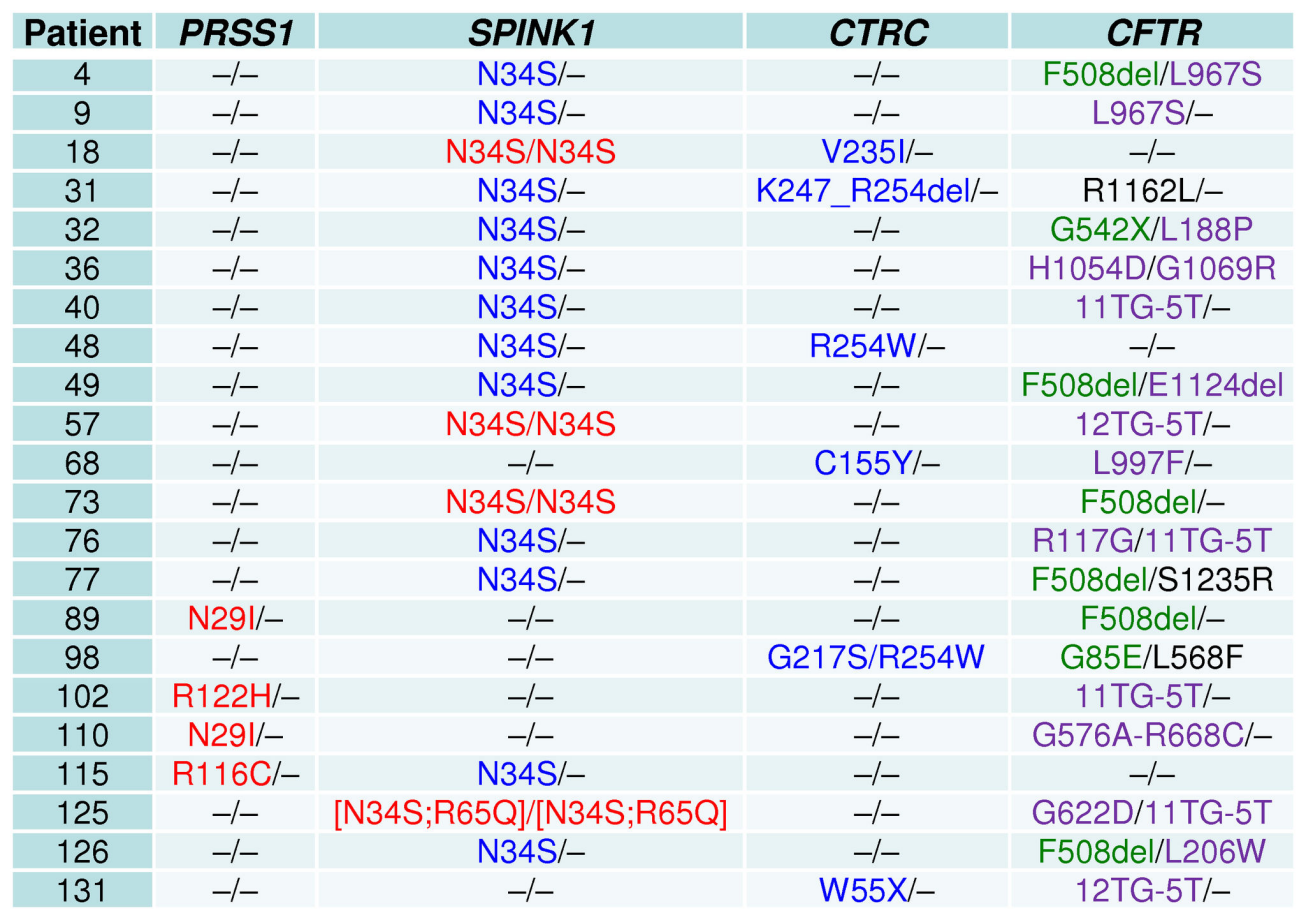
22 ICP patients displaying evidence of a digenic inheritance pattern. In *PRSS1*, *SPINK1* and *CTRC*, the variants or genotypes classified variously as causative, contributory or neutral are given in red, blue and black, respectively. In *CFTR*, the alleles classified individually as severe, mild or associated with CFTR-related disorders, and neutral are in green, violet and black, respectively. See text for details regarding the definition of causative, contributory and neutral *CFTR* genotypes and gene-gene interactions.

Since we aimed to be conservative in our estimate of the major genetic cause of ICP, we excluded synonymous, intronic and 5'- or 3'-untranslated region variants in the four genes from consideration except where there was persuasive evidence of a functional consequence. All remaining variants (available in an Excel format in Supplementary Table **S1**) will be discussed in the context of their associated genes, followed by a consideration of potential gene-gene interactions in accordance with the principles set out in Figure **1**. In particular, the 22 patients found to exhibit gene-gene interactions are described in Figure **2**.

### Rare causative PRSS1 mutations account for 9.1% of the ICP study group

Heterozygous variants in the *PRSS1* gene were identified in 25 of the 253 ICP patients (Supplementary Table **S1**). Of these, p. P36R was found in a single male subject; this variant had been previously reported in a French female ICP patient but not in 400 French healthy controls [29]. However, a recent functional analysis has suggested that this variant may not be of any pathological relevance [30]. p. E79K was also found only once; this variant has been previously reported in several studies [29,31–34] whilst functional analysis has suggested a possible pathogenic mechanism involving trans-trypsinogen activation [32]. However, p. E79K was found to be present in both French and Brazilian controls, with allele frequencies of 0.25% [29] and 0.33% [31], respectively. From a conservative standpoint, and given the general rule that the representation of gain-of-function mutations in a given gene is likely to be very limited, these two missense mutations were classified as neutral alleles.

By contrast, neither the *PRSS1* gene duplication (× 4) and triplication (× 9) copy number mutations, nor the p. N29I (× 4), p.R122H (× 3), p.R122C (× 1), p.R166C (× 1), and p. A16V (× 1) missense mutations have ever been described in unrelated healthy controls. Moreover, each lesion was either presumed (in the case of copy number mutations) or experimentally shown (in the case of missense mutations) to have a plausible pathogenic mechanism leading to chronic pancreatitis (for references, see 35). Taken together with the key role of *PRSS1* in the aetiology of chronic pancreatitis [8], all these *PRSS1* mutations could be regarded as causing the disease in their respective carriers (N = 23), accounting for 9.1% of the 253 ICP patients screened (Table **S1**).

### *SPINK1* variants either caused or contributed to ICP in 6.7% and 9.1% of cases respectively

Based on the observation that some of the most severe *SPINK1* alleles (e.g. micro-deletions and splice site mutations) were reported in families with hereditary pancreatitis, we concluded that haploinsufficiency of the *SPINK1* gene is sufficient to cause the disease [8]. In line with this postulate, partially functional *SPINK1* alleles can be assumed to predispose to the disease in a dosage-dependent manner. Such a dosage-dependent influence may however often have been masked by the variable penetrance and expressivity of the condition.

Variants in the *SPINK1* gene were found in 42 of the 253 ICP subjects screened (Table **S1**). Of these, p. P55S, which was found in three cases, is an unambiguous neutral polymorphism; it was found both in cases and controls with comparable frequencies [13,19,36] and exhibited no functional effect when characterized *in vitro* [37,38].

p. N34S, in the simple heterozygous state (e.g. patient 4, Figure **2**), was present in 9.1% (N = 23) of the 253 studied ICP cases [p. N34S may not be of functional significance *per se* and may simply represent a marker of its associated haplotype [39,40]]. Since heterozygous p. N34S is present in ~0.75% of the French population [41], the p. N34S-associated haplotype would appear to increase the risk of ICP by ~12-fold. This is consistent with the two largest and most relevant studies to date, which reported a 10-15 fold increased risk for chronic pancreatitis [19,42].

Homozygous p. N34S (e.g. patient 18, Figure **2**) was present in 5.5% (N = 14) of the 253 ICP cases. By way of comparison, combined data from eight studies performed in Europe and the United States indicate that ~3.6% of chronic pancreatitis patients are homozygous for p. N34S [18]; the corresponding proportion from the recent Rosendahl study was 2.6% [19]. In addition, in the Rosendahl study, the frequency of homozygous p. N34S in the <20 years group was higher than in the >20 years group [3.1% (13/421) vs. 1.7% (4/239)], although this difference was not statistically significant owing to the small sample size.

We may assume that homozygosity for p. N34S serves to double the functional effect of a single heterozygous p. N34S allele. However, this quantitative addition may lead to a qualitative change in terms of phenotype expression were it to reduce the *SPINK1* expression below a threshold level that is sufficient to cause the disease. Another important point is that, to date, no p. N34S homozygote has ever been reported from a control population [18,19,42]. Given the already >10-fold increased risk conferred by the heterozygous p. N34S variant, we believe that it is not unreasonable to classify homozygous p. N34S as disease-causing. By way of reference, according to the rare allele model of complex disease, rare variants (allele frequency typically <1% in the normal population) that confer an elevated risk two-fold or more above background, are generally held to be the main cause of the disease in question [43].

Three known rare *SPINK1* mutations, p. M1 [13]? , IVS2+1G > A [21] and IVS3+2T>C [13], each of which was found once in the current study, were deemed to be disease causing by virtue of their predicted functional consequences. These three mutations (and other presumed or experimentally demonstrated loss-of-function mutations) in the *SPINK1* gene were almost invariably found in patients rather than controls [8,19].

The aforementioned p. M1? variant was identified in *trans* with p. N34S (i.e. a compound heterozygote) in patient 45 (Table **S1**). In addition, one of the 14 p. N34S homozygotes was in fact doubly homozygous for p. N34S and p. R65Q (patient 125, Figure **2**). p. R65Q is itself of pathogenic relevance, reducing SPINK1 protein expression to 40% of normal [37,38]. Since heterozygous p. M1? and homozygous p. N34S have both been regarded as disease-causing, these combinations do not alter the carrier frequency with respect to disease-causing *SPINK1* genotypes.

In summary, *SPINK1* variants caused the disease in 6.7% (N = 17) of the studied cases and could have contributed to disease development in an additional 9.1% (N =23) of cases. Taken together, pathological *SPINK1* variants were found in 15.8% of ICP cases (Table **S1**).

### Rare CTRC genotypes contributed to the development of ICP in 4.3% of cases

*CTRC* has conventionally been regarded as being the least important gene in terms of a genetic predisposition to chronic pancreatitis, as compared with *PRSS1* and *SPINK1* [8]. This view received further support from two recent developments. First, some of the *CTRC* variants characterized by a complete or virtually complete functional loss of the affected alleles, exemplified by p.K247_R254del and p. G217S, have been reported in unrelated healthy controls [44]. Second, whereas a heterozygous *CTRC* whole gene deletion was found in *trans* with other genetic predisposing alleles in two subjects with familial chronic pancreatitis, a different homozygous *CTRC* whole gene deletion was identified in a patient with asymptomatic ICP [45]. Employing the functionally null *CTRC* allele, p.K247_R254del, which increases the risk of ICP 6.4-fold [44], as a reference (N.B. the heterozygous *SPINK1* p. N34S allele confers a >10-fold increased risk), any loss-of-function variants in the *CTRC* gene may at most be interpreted as disease-predisposing.

Variants in the *CTRC* gene were found in only 14 subjects in this study (Table **S1**). Of these, three missense mutations, p.K172E, p.R162H and p.M200V, each found only once, were classified as neutral variants on the basis of functional analysis [44]. All the other variants were either clearly pathogenic (p. W55X) or experimentally demonstrated (p.R254W, p.V235I, p.K247_R254del, p. A37T, p. C155Y, p. G217S, and p.G217R) loss-of-function variants. For reasons of simplicity and from a conservative viewpoint, all these variants, irrespective of (i) their occurrence in the heterozygous (e.g. patient 18) or compound heterozygous (e.g. patient 98, Figure **2**) state and (ii) being classified as high risk or moderate-to-low risk variants by Beer and colleagues [44], were here invariably classified as being disease contributory. Consequently, *CTRC* susceptibility variants were found to be present in 4.3% (N = 11) of the patient group (Table **S1**).

### *CFTR* genotypes contributed to, or caused, ICP in ~24% and 4.0% of cases, respectively

A diverse range of *CFTR* variants/genotypes were found in a total of 99 ICP cases (Table **S1**). As indicated in Figure **1**, these variants were first divided into three categories on an individual basis. For example, F508del, L967S and R1162L (Figure **2**) fell into the A (cystic fibrosis-causing severe alleles), B (cystic fibrosis mild alleles and those associated with CFTR-related disorders [7]) and C (the remaining *CFTR* alleles) categories, respectively. In general, we treated those alleles of unknown significance conservatively by placing them into the C category. Of particular note, p. R75Q was placed in category C on the basis that it was not overrepresented in our patient cohort [4.7% (12/253)] as compared with a sample of 514 French controls [4.9% (25/514)]. This finding concurs with that of the recent Rosendahl study [19] but differed from that of Schneider et al. [46]. In the latter study, p. R75Q was reported to be overrepresented in patients as compared to controls (16% vs. 5.3%). Contrary to the findings of the Schneider study [46], we did not find any preferential co-occurrence of *CFTR* p. R75Q with *SPINK1* p. N34S; only two of our seven heterozygous *CFTR* p. R75Q patients also carried a heterozygous *SPINK1* p. N34S (patients 19 and 130, Table **S1**).

Based on our current knowledge of the *CFTR* genotype/phenotype relationship, *CFTR* genotypes in each of the 99 subjects (i.e. both the two alleles were taken into consideration) were classified as causative, contributory or neutral in accordance with the combinations of the A, B and C category alleles (Figure **1**). For example, the compound heterozygote in patient 4 was regarded as being causative because it comprised a category A allele plus a category B allele; the genotypes in patients 73, 9 and 36 were regarded merely as contributory because they comprised a simple heterozygous category A allele, a simple heterozygous category B allele and two category B alleles, respectively; by contrast, the genotype in patient 31 was held to be neutral because it comprised a simple category C allele (Figure **2**). In accordance with this classificatory scheme, 4.0% (N = 10) of the patients carried a causative *CFTR* genotype whilst 23.7% (N = 60) carried a contributory genotype (Table **S1**).

### Gene-gene interactions were found in ~9% of ICP patients

122 ICP cases were found to carry at least one contributory or causative genotype as assigned in this study (indicated by x or xx in column F, Table **S1**), accounting for 48.2% of the 253 studied patients. Of these 122 cases, 100 carried a single pathological genotype (indicated by 1, 2, 3 or 4 in column G, Table **S1**), 22 carried two pathological genotypes in two different genes (termed digenic genotypes; indicated by 12, 14, 23, 24 or 34 in column G, Table **S1**; see also Figure **2**). All of the 22 latter cases, accounting for 8.7% of the 253 ICP patients, comprised at the most only one disease-causing genotype. Here it is pertinent to mention that in the Rosendahl study [19], trans-heterozygotes were found in 43/660 (6.5%) patients and 3/1667 (0.2%) controls (*P* <0.0001), OR 38.7; 95% CI 12 to 125.1).

Of the aforementioned 22 cases, 18 involved a pathological *CFTR* genotype (Figure **2**), presumably due to the relatively high frequency of pathological *CFTR* genotypes in the patients. Of the 18 cases involving a pathological *CFTR* genotype, 12 harboured a pathological *SPINK1* genotype, 3 a pathological *PRSS1* genotype, whilst the remaining 3 also harboured a pathological *CTRC* genotype (Figure **2**). It should be noted that in the 12 *CFTR*/*SPINK1* digenic pathological genotypes, the *SPINK1* partner was invariably the p. N34S variant (heterozygous × 10; homozygous × 3) (Figure **2**).

**Figure 3:**
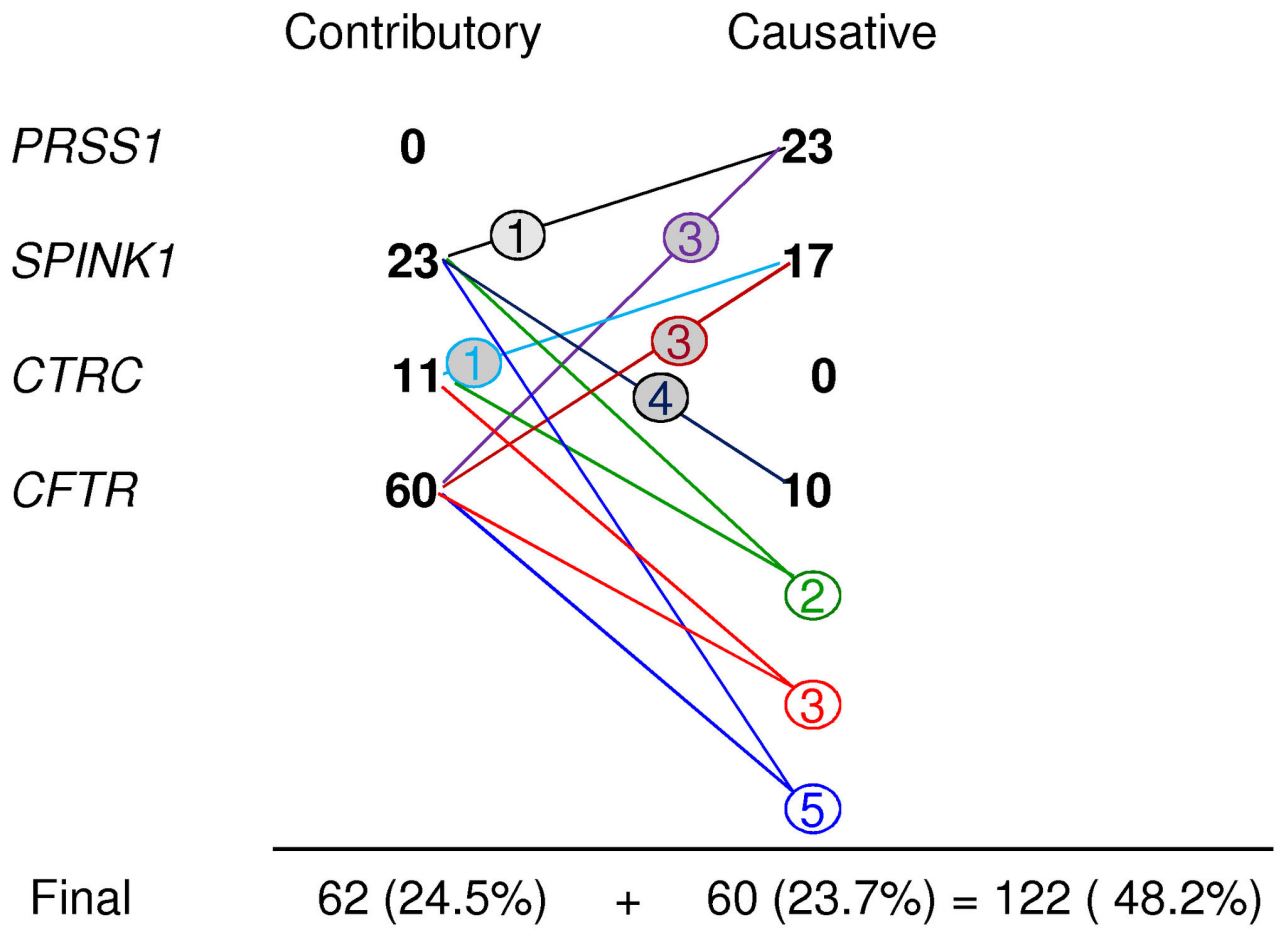
An overview of the major genetic cause of ICP resulting from the analysis of the four major pancreatitis genes in 253 young French ICP patients. Numbers in bold indicate the contributory and causative variants/genotypes assigned in the context of the *PRSS1*, *SPINK1*, *CTRC* and *CFTR* genes, individually. Numbers in shaded circles indicate evidence of a digenic inheritance pattern comprising a causative genotype in a gene modulated by a contributory genotype in another gene. Numbers in non-filled circles indicate two contributory genotypes in two distinct genes (refer to Figure **2**); these digenic genotypes were further assigned as causative by taking into consideration the presumed additive or potentially synergistic effects of their component genotypes. The final counts of contributory, causative and contributory plus causative genotypes are also provided.

Of the 22 digenic pathological genotypes, 12 comprised a disease-causing genotype and a contributory genotype (patients 4, 18, 32, 49, 57, 73, 89, 102, 110, 115, 125 and 126; Figure **2**). The remaining 10 cases comprised two contributory genotypes (patients 9, 31, 36, 40, 48, 68, 76, 77, 98 and 131; Figure **2**). These 10 cases were further assigned as causative due to the presumed additive or potentially synergistic effects of their component contributory genotypes involving two genes. This approach increased the number of causative genotypes to 60, representing 23.7% of the 253 ICP patients screened. Finally, contributory genotypes were found in an additional 24.5% (N = 62) of cases. Taken together, up to 48.2% of the studied ICP patients were found to display evidence of a genetic basis for their pancreatitis (Figure **3**).

### Conclusions and perspective

We systematically screened a large and fairly homogeneous sample of ICP patients for both conventional types of mutation and gross genomic rearrangements involving the four most extensively studied chronic pancreatitis-causing or -predisposing genes. Although the extensive locus and allelic heterogeneity of the disease presents a major challenge, we have devised a classificatory scheme that should serve as an invaluable aid in dissecting the complex genetic determinants of ICP. This notwithstanding, we would like to make three points. First, the proposed variant classification scheme should not be regarded as definitive and is certainly likely to be subject to modification and improvement over time in the light of new genetic, epidemiological and functional data as they emerge. Second, the particular proportions we obtained in the 253 young French patients may not be extrapolable to ICP patients from other populations and different age groups. For example, trypsinogen duplication and triplication copy number mutations were detected in 5.1% (N = 13) of the 253 cases; to date, they have not been reported by other groups. Whether this is due to the absence of such events in other populations or simply due to the fact that they have not been sought in a methodical fashion is unclear. Third, and relevant to the ‘conservative’ nature of the study, it is worth pointing out that the intronic regions of the four major genes were not screened for mutations. Despite these limitations, we have demonstrated that a prerequisite for understanding the genotype-phenotype relationship in a complex disease such as ICP is not a simple variant number count but rather an overall assessment, made on an individualized basis, of the net effect of an oligogenic variant profile on the eventual risk of disease.

## Supporting Information

Table S1 **Variants/genotypes reported in the current study.**
In columns B, C and D, the variants or genotypes classified variously as causative, contributory or neutral are given in red, blue and black, respectively. In column E, the CFTR alleles classified individually as severe, mild or associated with CFTR-related disorders, and neutral are in green, violet and black, respectively. In column F, x and xx indicate contributory and causative genotypes, respectively, with gene-gene interactions being taken into consideration. In column G, 1, 2, 3 and 4 refer to pathological PRSS1, SPINK1, CTRC and CFTR genotypes, respectively. See text for details.
(XLS)
